# Social capital's impact on COVID-19 outcomes at local levels

**DOI:** 10.1038/s41598-022-10275-z

**Published:** 2022-04-21

**Authors:** Timothy Fraser, Courtney Page-Tan, Daniel P. Aldrich

**Affiliations:** 1 Political Science Department, Northeastern University, Boston, MA 02115 USA; 2 Security and Emergency Services Department, Embry-Riddle Aeronautical University, Daytona Beach, FL 32114 USA; 3 Security and Resilience Program, Department of Political Science, School of Public Policy & Urban Affairs, Northeastern University, Boston, MA 02115 USA

**Keywords:** Risk factors, Environmental social sciences, Socioeconomic scenarios, Public health

## Abstract

Over the past thirty years, disaster scholars have highlighted that communities with stronger social infrastructure—including social ties that enable trust, mutual aid, and collective action—tend to respond to and recover better from crises. However, comprehensive measurements of social capital across communities have been rare. This study adapts Kyne and Aldrich’s (*Risk Hazards Crisis Public Policy* **11**, 61–86, 2020) county-level social capital index to the census-tract level, generating social capital indices from 2011 to 2018 at the census-tract, zipcode, and county subdivision levels. To demonstrate their usefulness to disaster planners, public health experts, and local officials, we paired these with the CDC’s Social Vulnerability Index to predict the incidence of COVID-19 in case studies in Massachusetts, Wisconsin, Illinois, and New York City. We found that social capital predicted 41–49% of the variation in COVID-19 outbreaks, and up to 90% with controls in specific cases, highlighting its power as diagnostic and predictive tools for combating the spread of COVID.

## Introduction

Why do some communities experience greater outbreaks of COVID-19? As of the time of writing on February 1, 2022, COVID-19 has led to at least 74.3 million cases and 884,300 known deaths in the US, and over 5.67 million deaths worldwide^1,2^. These outcomes represent major challenges to communities’ *resilience*, referring to residents’ “capacity to adapt existing resources and skills to new situations and operating conditions”^3^. Past studies highlighted that pandemic outcomes, like many disaster resilience outcomes, depend on more than the capacity of governments and health care workers to respond^4,5^ and the social vulnerability of residents by race, class, age, and gender^6–9^. However, crisis outcomes *also* correlate with the strength of community resources such as social infrastructure accessible to members^10–13^. Social capital—the social ties that enable trust, reciprocity, and collective action^14^—serves as a key resource residents can draw upon before, during, and after crisis to ensure that they obtain mutual aid from family, friends, and neighbors and also gain access to key public or semi-public goods from lawmakers^15,16^. Past scholars measured social capital using regional level responses from the Global Social Survey^17^, custom neighborhood surveys in disaster zones^18^, and aggregate measures at the county level^19^. However, the impact of COVID-19 varies widely by neighborhood. Scholars and local officials need a clearer measure of social ties at increasingly hyperlocal levels.

This study introduces new measures for social capital for every census tract, zipcode, and county subdivision in the United States from 2010 to 2018. These indices characterize the strength of bonding, bridging, and linking social capital, as well as overall levels of social capital. Rather than reinventing the wheel, these measures draw directly from the methodology of Kyne and Aldrich’s^19^ validated county level Social Capital Indices, extending it to the census tract level. Then, we average these census tract measures up to the zipcode and county subdivision level to create broad coverage for measuring social capital. Finally, we apply these indices to predict COVID-19 outcomes in different regions of the United States, showing frequent associations with COVID-19 test positivity rates.

This study makes three main contributions to the literature. First, we extend Kyne and Aldrich’s^19^ county level measures of social capital to the census tract, zipcode, and county subdivision, enabling comparison of tens of thousands of communities in terms of bonding, bridging, and linking social ties. This adds an important resource to scholars and policymakers involved in disaster and pandemic response efforts, easily paired with the CDC’s Social Vulnerability Index^20^ and the Baseline Resilience Indicators (BRIC)^21^.

Second, we find that these local level measures of social capital are closely correlated with a key diagnostic measure of COVID-19 spread, the percentage of COVID-19 tests returned positive. Our models accounted for up to 90% of the variation in COVID-19 spread. This builds on recent research that suggests that social capital helps residents adopt new behavioral norms like social distancing and masking and reduce COVID-19 spread^10–13^, while verifying it with a measure that adjusts for testing capacity.

Third, we find that social capital has divergent effects depending on the type of social capital present in a community and the local context of each city or region. First, each kind of social capital index produces strong, significant associations with test positivity rates in multiple cases, and bonding social capital is linked to reduced COVID-19 spread in regions at large, matching past studies^10,12,22^. However, in specific cities the effect of bonding social capital frequently changes: communities with strong bonding ties promote COVID-19 spread through insular social networks, while strong bridging ties help reduce that spread in urban environments. This matches results from disaster studies, which highlight that while bonding social capital can help *some* communities, bridging ties aid *most* communities by fostering trust, reciprocity, and pro-social behavioral changes across different racial, ethnic, religious, and political lines^23–25^.

## Literature review

Since the COVID-19 pandemic reached the US in early 2020 (or arguably earlier^26^), local, state, and federal government agencies have made tracking key COVID-19 outcomes a top priority, including case rates, death rates, test positivity rates, excess deaths rates, and vaccination rates. States governments across the country have digitized measurements of these new pandemic resilience outcomes, creating online dashboards for health officials to more easily communicate with policymakers, the press, and the public, and to engender greater trust and transparency in crisis information flow. While these dashboards provoked early pushback from state government officials in denial about the scope of the crisis, they have become powerful tools for highlighting hotspots of vulnerability, as it became clear quickly that communities of color, working class neighborhoods, front line workers in crowded areas, the aged, and residents with pre-existing conditions faced high health or financial risks from COVID. While some dashboards (see for example this Alabama dashboard^27^), embraced the CDC's Social Vulnerability Index, other key indicators of disaster resilience have been conspicuously absent in pandemic planning dashboards and data monitoring. Below, we review past explanations from disaster scholarship for pandemic resilience, including mobility, health care capacity, quality of health, governance capacity, political partisanship, and social capital.

First, some communities might see higher rates of COVID-19 spread due to greater *population mobility*. While past crises, like SARS and the Avian flu^28,29^, highlighted that travel can spread viruses across borders, this was complicated in the ongoing pandemic by the fact that many persons infected with COVID-19 remain asymptomatic^30^. Among symptomatic patients, 95% of individuals manifest symptoms between 2 and 11 days after contact with COVID, with a median of 5 days^31–33^. Recent studies of COVID-19 spread have shown that lockdowns, compulsory^34^ or not^35^, greatly reduced mobility and likely altered the course of the epidemic.

Second, communities with *better staffed and funded health care systems* might respond more adroitly to the pandemic. The capacity of health care systems, measured by better hospital quality and larger physician workforces^36,37^, has been linked to better response quality both before^4^ and during COVID-19^38^. Similarly, more *capable governments* which can effectively allocate resources, purchase appropriate protective gear, manage information flow, enforce lockdowns, and ensure high shares of residents with health insurance may see better health and pandemic outcomes^34,39,40^. Meta-analyses over the last 2 years have highlighted common comorbidities of COVID-19^41–43^. For example, Shakaib and colleagues’ meta analysis^43^ ranked comorbidities by prevalence as (1) *hypertension* (~ 28.6% of patient deaths), (2) *heart conditions and stroke* (14.6% and 8.3%); *diabetes* (13.2%), smoking and chronic lung disease (8.1% and 3.2%), chronic kidney and liver diseases (7.2% and 2.7%), and being immunocompromised (4.8%). Obesity also co-occurs frequently with severe cases; in studies of New York City hospital patients with severe cases of COVID-19, 35.8% to 60.6% reported obesity^44,45^; obese COVID-19 patients tend to see 37% higher rates of death during hospitalization and risk of contracting pneumonia^46^.

Third, communities may see different mobility patterns depending on their levels of *political partisanship*. Several notable Republican national and state election officials consistently downplayed the COVID-19 pandemic, despite multiple superspreader events at the White House in 2020^47^. These sentiments played out among the general public: By mid-July 2020, just 45% of Republican voters ranked COVID-19 as a major threat to the country compared to 85% of Democrats^48^. Multiple studies have highlighted that Republican elected officials and voters have been less likely to adopt social distancing and other COVID-19 prevention measures than their Democratic peers^49–51^.

Fourth, some communities see worse resilience to crisis due to their levels of *social vulnerability*^6^. In both pandemic and disaster studies, socially vulnerable residents fare worse in terms of resilience to crisis due to employment challenges, limited mobility, and the fear of discrimination or retribution from neighbors or local authorities. Groups more socially vulnerable to disaster including women and single parents^47^, families facing unemployment or poverty^52^, the elderly^53^, the LGBTQ+ community^54^, the disabled^55^, and racial, religious, or ethnic minorities^56^. During COVID, Black Americans have overwhelmingly felt a higher burden^7–9,57^, with 1 in 1000 African Americans dead from COVID-19^58^ at the same time as crushing financial loss, with over 40% of Black-owned businesses in the US closed permanently this past year^59^. However, previous research^60^ has found that state and local governance has made a difference in the pandemic for socially vulnerable communities. Counties considered highly vulnerable based on the CDC’s Social Vulnerability Index that enacted face mask requirements, gathering restrictions, and stay-at-home orders experienced reductions in the average number of COVID-19 deaths, compared to similarly high vulnerability counties that did not enact these non-pharmaceutical interventions.

Finally, some communities, even facing high social vulnerability or weak health care response, have managed better pandemic outcomes than others due to their *social capital*. Social capital—the social ties that bind together a community and enable trust, reciprocity, and collective action^14^—serves as a key resource for residents responding to disaster^24^, closely correlated with health outcomes^16^ and recovery outcomes after crisis^18,61^. Recent survey and aggregator level research has demonstrated that residents and communities with stronger social capital were more likely to socially distance^62^, see fewer cases and fatalities^10,11^, and slower spread of COVID-19^12^. However, disaster studies acknowledge the Janus-faced nature of social capital, in which social capital can generate both positive and negative outcomes depending on the type of social ties fosters^63^. Indeed, recent studies of social capital highlight that vertical social ties to government officials are especially key, relative to others^22,64^.

Social capital comes in three forms: bonding, bridging, and linking social capital^65^. Bonding social capital refers to close ties between members of the same social circles, facilitating trust and mutual aid among friends and family members^23,66^. Bridging social capital describes association ties between members of different social groups, built through workplaces^67^, unions^68^, volunteering^17^, sports clubs^14^, and local associations^69^. Bridging ties are the lifeblood of democracy, helping residents build shared stake in their community and enabling close cooperation during and after crisis^24,69^. Finally, linking social capital refers to vertical ties connecting residents to local, state, and national authorities^15^. These linking ties help residents access key public goods from elected officials and instill trust in government^25,70^, which has been linked to better public compliance with public health protocols during outbreaks^71^, including SARS^72^, Ebola^73^, and COVID-19 crises^64^. We might expect that linking and bridging ties aid COVID-19 response, while bonding, insular ties might limit the spread of information and lead to less resilient response under certain conditions^63^.

## Results

This study examines what kinds of communities experience greater outbreaks of COVID-19, and to what degree local level measures of social capital and vulnerability can predict those outbreaks. As our main outcome of interest, this study investigates *COVID-19 test positivity rates* (the percentage of tests that come back positive)^74^, approximating the spread of COVID-19, widely adopted in past studies of community spread^75–79^. While case incidence rates are biased because some states test more than others, test positivity rates adjust for the number of total tests performed in an area. They are also now widely available in some cities and regions at extremely local geographies—the census tract, zipcode, and county subdivision levels being the focus of this paper.

**Figure 1:**
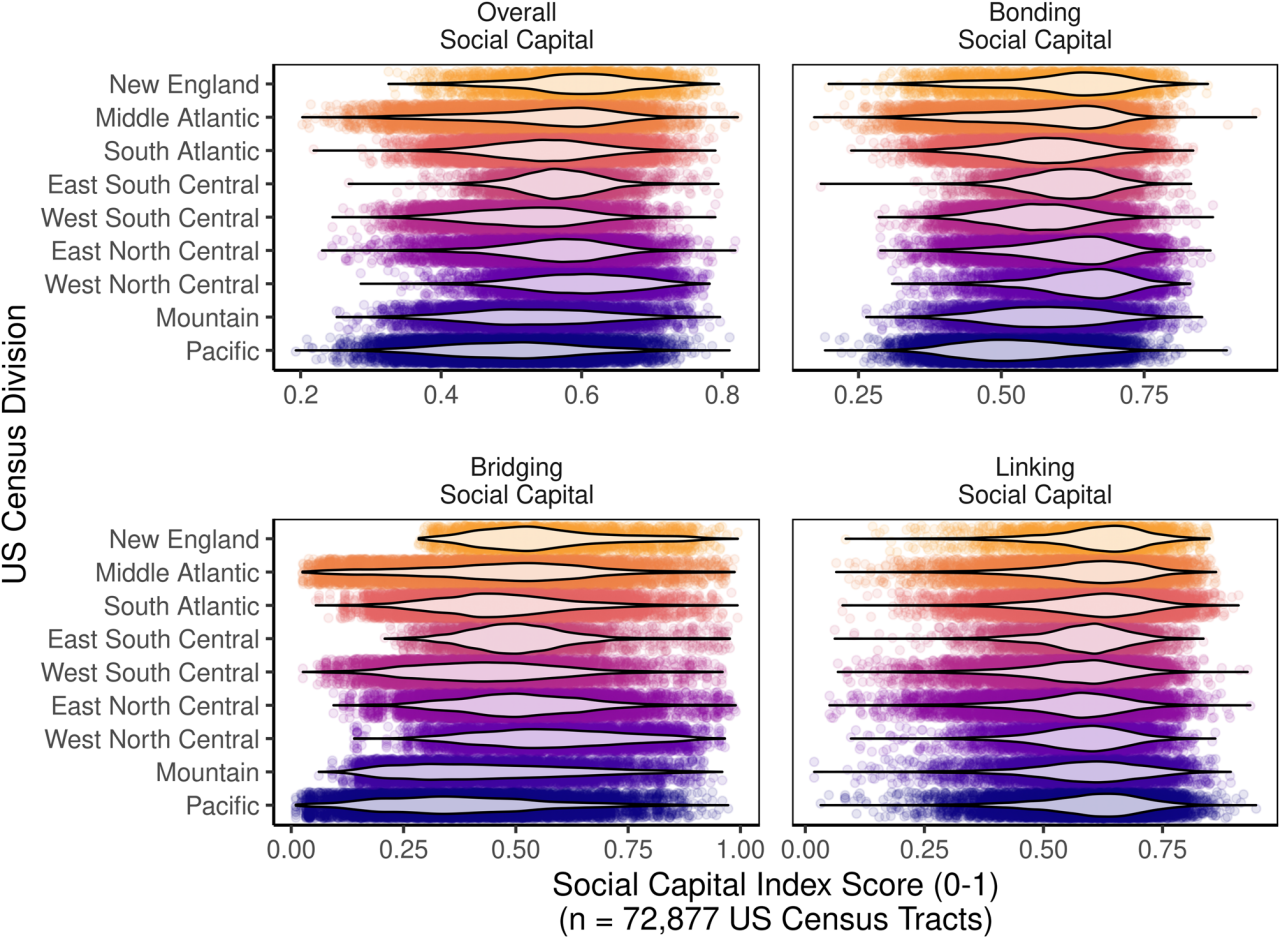
Geographic distribution of social capital indices at the census-tract level. Violins depict distributions of social capital indices for each of the 9 US Census-bureau designated geographic divisions in the US.

This study adapts existing, validated methodologies for measuring social capital at the county level^19^ to new measures at the census tract, zipcode, and county subdivision levels. This study generated social capital indices at the census tract, zipcode, and county subdivision levels, and applied them to case studies within and across counties in Wisconsin, New York, Illinois, and Massachusetts. Each index was measured at the census tract level, and their distributions are visualized across US Census Divisions in Fig. 1 to demonstrate their degree of geographic variation.

**Figure 2:**
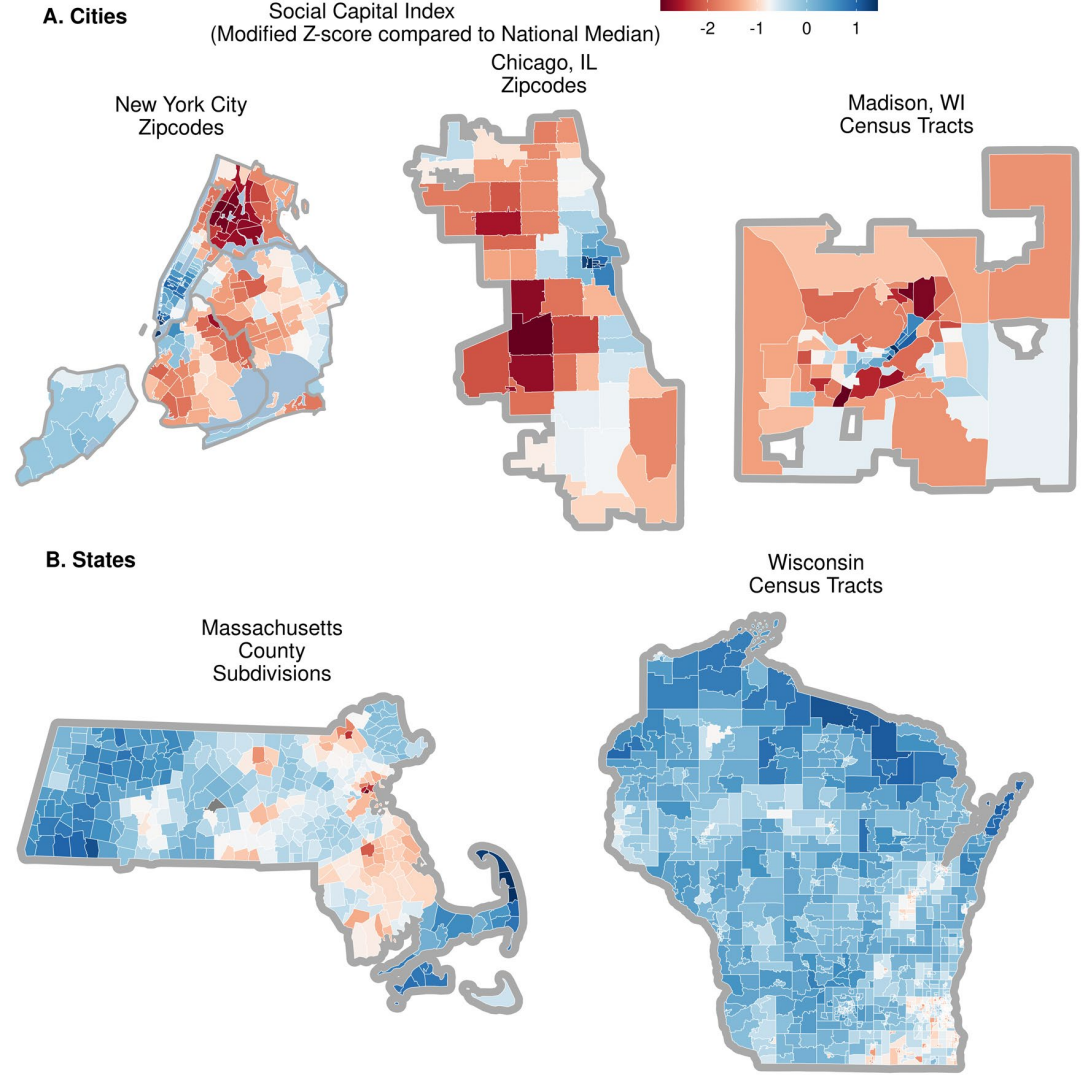
Variation in overall social capital among case studies. This fiugure depicts communities as zipcodes, census tracts, or county subdivisions. Shading represents social capital, measured as a modified Z-score, showing distance from the national median standardized by the median absolute difference (MAD). White indicates national median level of social capital. Blue shows MAD-calculated standard deviations above median, while red depicts below median. Maps made in R (version 4.0.3) using the sf package (version 1.0-6)^81^.

As shown in Fig. 2, these case studies demonstrated considerable variation in overall social capital compared to the national median, with urban areas showing especially troublingly low levels of social capital. Below we explore how this variation might shape resilience to COVID-19, summarizing findings from these models (Tables B1–B3). Model tables in the Supplementary Information appendix report the projected increase in log-test positivity rates given an increase of one standard deviation in the predictor; all effect sizes are comparable among predictors.

### Variation in COVID-19 spread explained

These models explained considerable amounts of variation in the outcome, as shown by the R^2^ statistics in Tables B1–B3. For models *across* counties in Table B1, our fully specified models explained 47–55% of variation in test positivity rates. However, even simpler versions explained high levels of variation too: Our models in Table B2 with just social capital measure predicted 41–49% (Models 1A, 2A, and 3A), increasing to 43–54% when paired with social vulnerability indicators (Models 1B, 2B, and 3B) (see “Methods”). This is considerable, especially considering the great differences between different kinds of counites involved (eg. all of Wisconsin). For models *within counties*, this includes anywhere from 8 to 89% of variation in the outcome, and 30–90% including fixed or random effects. The best explained cases were Brooklyn (90%), Queens (81%) and Manhattan (87%).*.*

**Figure 3:**
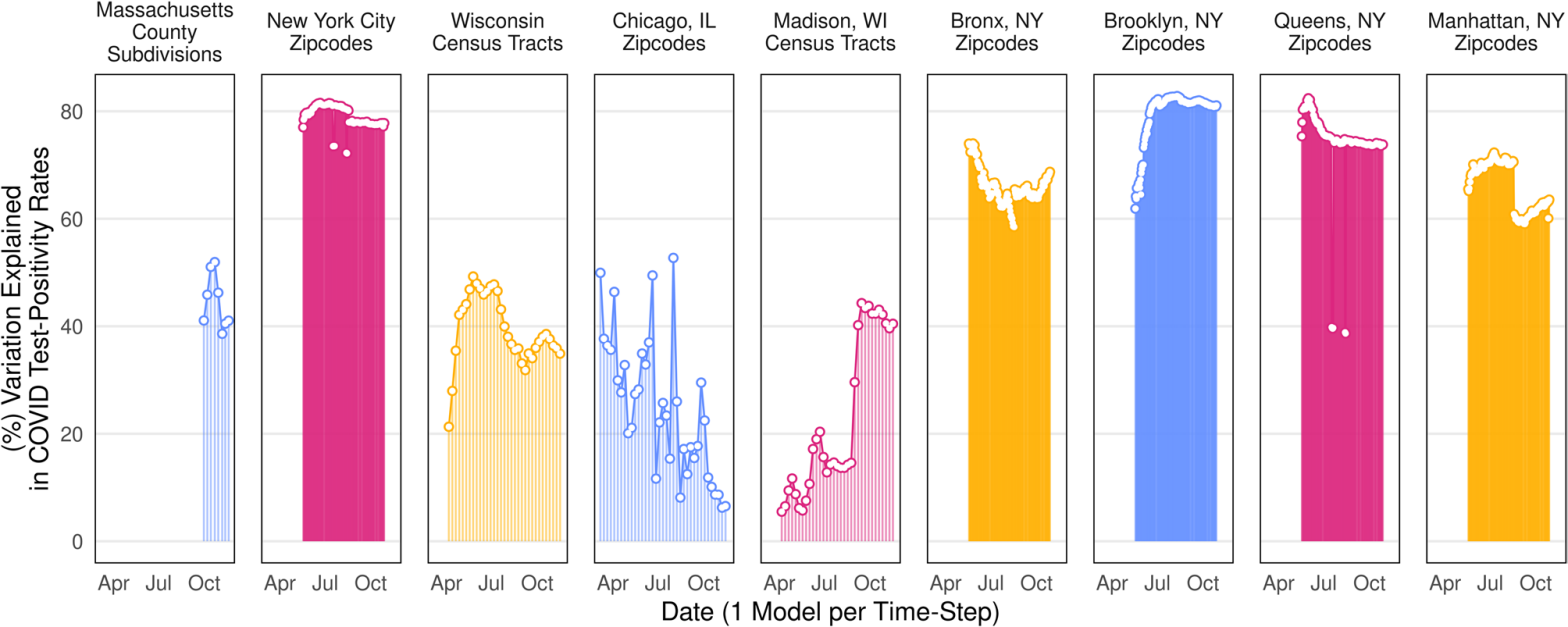
Variation explained by model covariates over time. Points depict variation explained (R^2^ statistic) from dozens of fully specified OLS models for each time-step. Indicates substantial variation explained by model covariates, separate from time.

Figure 3 contextualizes this by displaying daily versions of these models among each case study. Our New York City models explain the model variation (60–80% in most zipcodes), all other models explained anywhere between 5 and 55% of variation, each crossing at least 40% at some point in the study period. Even when regressed as dozens of individual models, in Fig. 3, we see that model covariates can explain quite high levels of variation over time.

### Effects of social capital on COVID-19 spread

**Figure 4:**
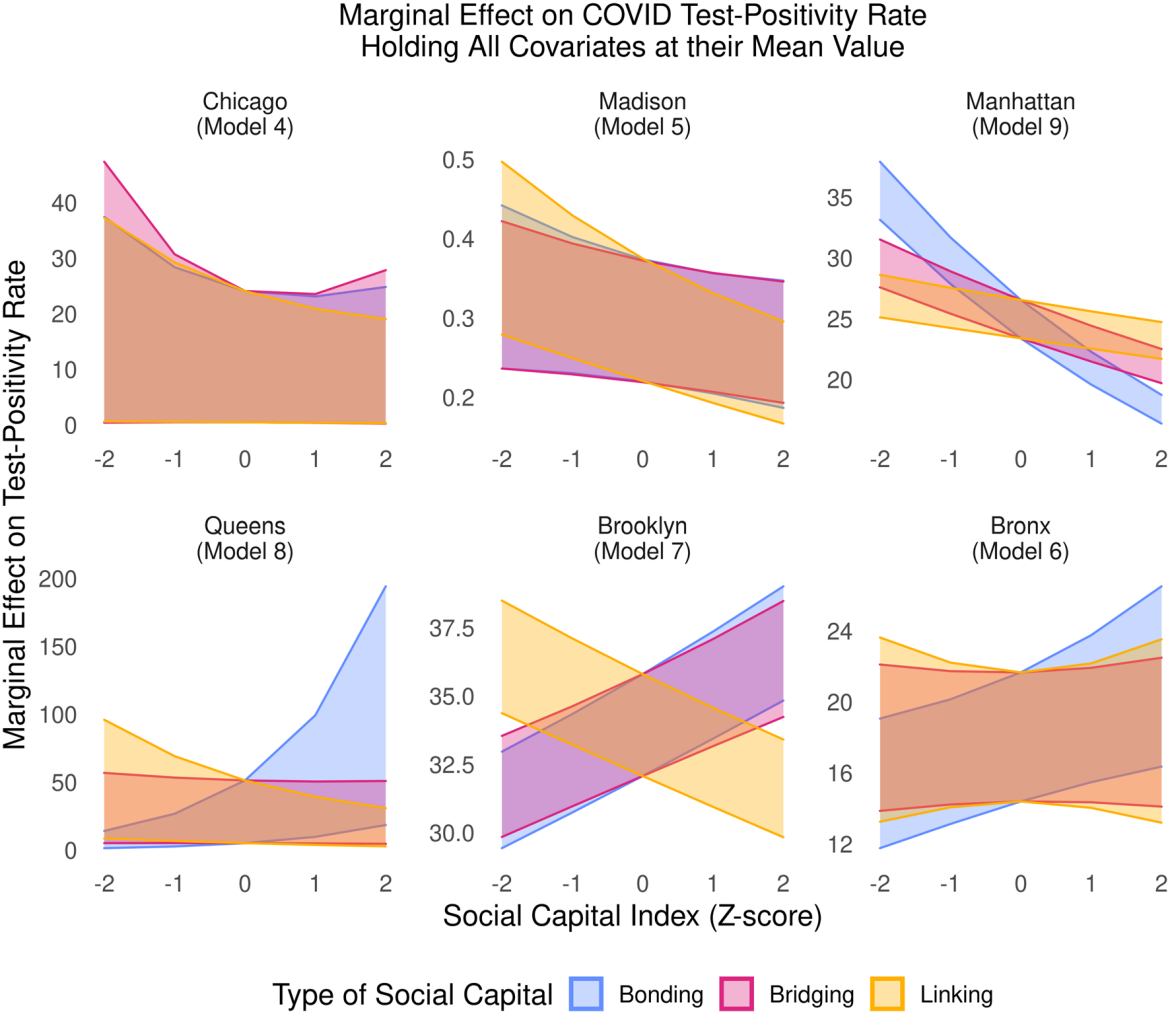
Marginal effects of social capital on COVID-test positivity rates. Bands depict marginal effect on test positivity rates as specified type of social capital varies by 2 standard deviations around the mean, holding all other variables at means and modes. Bands reveal varied effect of social capital subtypes depending on geography.

This analysis produced three general trends, highlighted in Fig. 4 using marginal effects, calculated holding all covariates at their means while varying each specific type of social capital. To ensure comparable effect sizes, all predictors in all models were rescaled as Z-scores before modeling, so that beta coefficients show the projected increase in the logged outcome as a predictor increases by 1 standard deviation from the mean (frequently called ‘standardized beta coefficients’). Similarly, Fig. 4 projects the test-positivity rate with 95% confidence intervals for an otherwise average community as each type of social capital independently increases from − 2 to 0 to 2 standard deviations from the mean level in each sample.

First, after adjusting for social vulnerability, health care capacity and conditions, governance capacity, and partisanship, we find that linking social capital, a close proxy for trust in government, is often negatively associated with test positivity rates. This occurs frequently across all *within county* models, and is shown by the consistently downward trending yellow bands in Fig. 4. Significant negative associations were found in Table B3 for Madison (beta = − 0.13, p < 0.001), Brooklyn (beta = − 0.04, p < 0.001), Queens (beta = − 0.28, p < 0.003), and Manhattan (beta = − 0.04, p < 0.001). Similar negative trends were found at large in Table B1 for Wisconsin (beta = − 0.13, p < 0.001) and New York City (beta = − 0.006, albeit with low significance at p = 0.823); the main exception was Massachusetts, which saw higher test-positivity rates (beta = 0.06, p = 0.058).

Second, we see mixed track records for bonding and bridging social capital. At large, bonding and bridging ties were linked to lower test-positivity rates: Our *across-county* models in Table B1 show that communities with strong bonding social ties saw lower test positivity rates, with significant associations for Massachusetts (beta = − 0.14, p < 0.007) and Wisconsin (beta = − 0.04, p < 0.001). Even moreso, communities with strong bridging ties saw significant negative associations for Massachusetts (beta = − 0.85, p < 0.001), New York City (beta = − 0.11, p < 0.003), and Wisconsin (beta = − 0.32, p < 0.001).

However, for *within-county* models in Table B3, the evidence for bonding and bridging ties diverges. Bridging ties were linked to lower COVID-19 spread in Madison (beta = − 0.05, p = 0.100) and Manhattan (beta = − 0.08, p < 0.001), with negative trends in Chicago with limited significance (beta = − 0.13, p = 0.696) and Queens (beta = 0.03, p = 0.756). Similarly, bonding ties were linked to lower COVID-19 spread in Manhattan (beta = − 0.18, p < 0.001), with less significant negative trends in Chicago (beta = − 0.103, p = 0.684) and Madison (beta = − 0.06, p = 0.153).

However, several cases diverged in Table B3. Greater bonding ties were linked to *greater* disease spread in Brooklyn (beta = 0.04, p < 0.001), the Bronx (beta = 0.08, p = 0.012), and Queens (beta = 0.66, p < 0.001). And greater bridging ties were linked to greater spread in Brooklyn (beta = 0.03, p < 0.001). Indeed, these communities were hard hit by COVID-19 early; Brooklyn is home to Spring Creek Towers, the subsidized housing development which suffered an outbreak leading their zipcode to face the highest death rate from COVID-19 in New York in the first 3 months of the outbreak, while the Bronx overall faced the highest rates for COVID-19 cases, hospitalizations, and deaths in the first 3 months^80^.

Yet this is not necessarily a contradictory finding. Past studies of disaster indicate that bonding social capital is fickle^24^; it can help family, friends, and members of the same social circle team up and share resources, but it can also promote insular social circles and inhibit the spread of quality information. In other words, we might expect that in historically marginalized communities where trust in local and national authorities is limited, that bonding social capital might not help these communities as much as bridging or linking social capital.

### Spatial differences in effects of social capital on covid-19 spread

**Figure 5:**
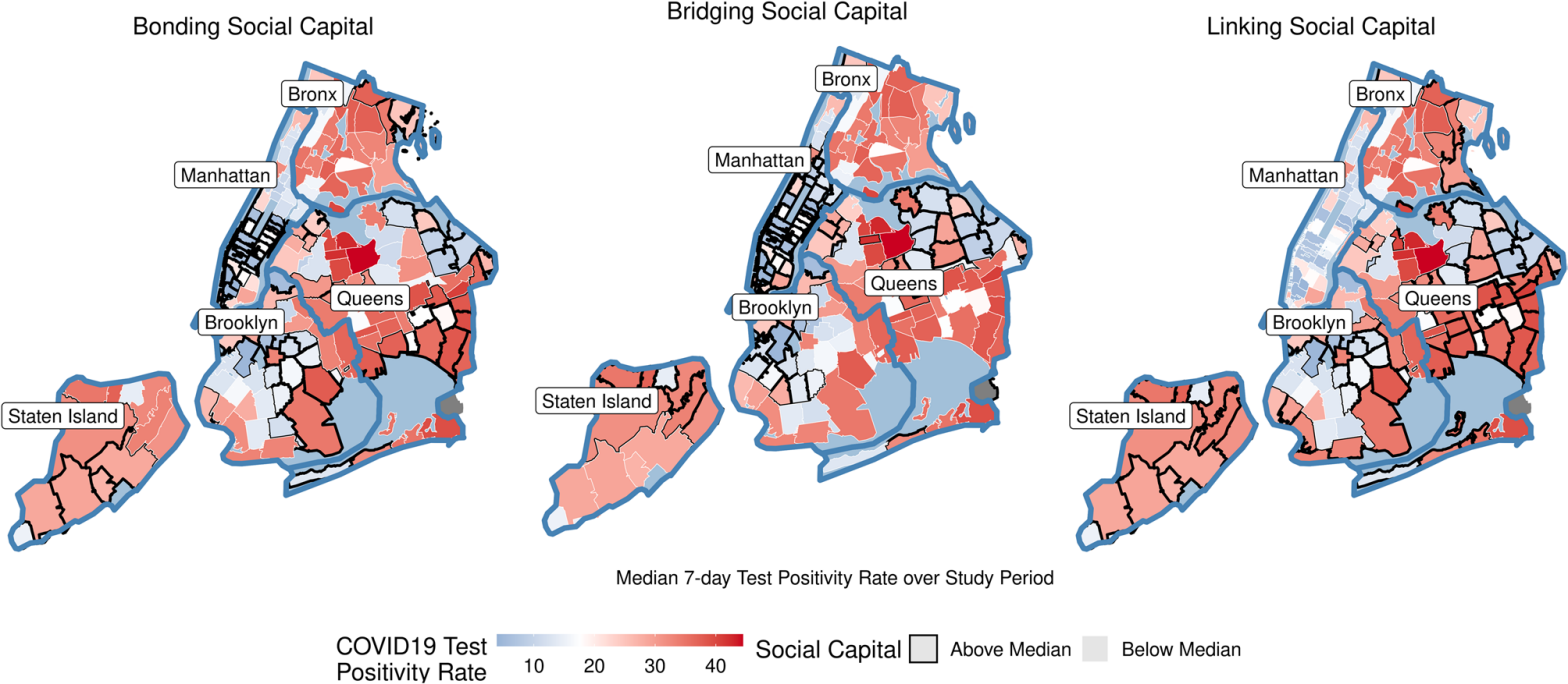
COVID-19 test positivity rates in New York City. Zipcodes of 5 boroughs based on whether above or below the median level of social capital in the NYC area. Blue lines depicting counties, a color scale depicting distance from the median COVID-19 test positivity rate (shaded white), averaged over time. Maps made in R (version 4.0.3) using the sf package (version 1.0-6)^81^.

Figure 5 highlights these trends in three panels, which highlight using black borders the zipcodes with the highest bonding (left panel), bridging (center panel), and linking social capital (right panel), compared to the COVID-19 test positivity rate, colored from blue (far below median) to white (median) to red (far above median). We see that bridging ties aided some parts of the city: many Bronx and Brooklyn zipcodes, with summarily low bridging social ties, saw high COVID-19 outbreak levels, while Manhattan zipcodes with strong bridging ties saw low COVID-19 outbreaks. In contrast, Staten Island experienced high test positivity rates on average, despite having strong bonding, bridging, and linking social capital, possibly leading to New York City’s overall skewed, positive effects of bridging and linking social capital on COVID-19 spread.

These findings about bonding and bridging social capital at first glance defy past findings in disaster studies, where after adjusting for bonding, bridging, and linking social capital, bridging ties, fostered through associations, typically encourage greater resilience to crisis than bonding, in-group ties. However, the nature of COVID-19 is especially challenging to bridging social capital. While linking social ties, as usual, help promote trust in public health and disaster response, bridging ties are usually indicated by connectedness to multiple different parts of society. In this case, that makes bridging residents in a community both capable of great good, reaching out to neighbors and supporting each other, but also capable of spreading the virus to new groups.

However, as highlighted by bridging social capital's negative effects on COVID-19 spread within the Manhattan, Madison, New York City, and Wisconsin at large, many communities with strong bridging social ties are developing important innovations to respond to COVID, ranging from virtual religious services and clubs to masked volunteer outreach and volunteering by teens out of school. For example, the Jewish Center located in Manhattan’s Upper West Side, which typically can accommodate 500 of its faithful, now can only accommodate 60 members who must pre-register and have their temperature taken on site before entering. Similarly, the Dar Al-Dawah mosque in Astoria, Queens now limits their in-person attendance to 64 people, requiring temperature checks and hand sanitization at the door for entry. Those who worship in-person are required to wear a mask and bring their own prayer rugs and place them in designated spots that allow for six feet of separation. Dar Al-Dawah has also offered additional services during religious holidays to accommodate higher demand for in-person, community worship^82^.

In other words, while strong bridging ties may not mean an automatic transition to resilience to the pandemic, they represent an important reservoir that city and health care officials can draw on to mobilize communities, provide aid, and address the "twin pandemics" of COVID's outright effects and its disproportionate effects on Black Americans.

## Discussion

This study measured bonding, bridging, and linking social capital for each census tract, zipcode, and county subdivision between 2010 and 2018 in the United States. Then, we applied these indices to predicting COVID-19 test positivity rates in Massachusetts, New York, Illinois, and Wisconsin, zooming in further to several urban areas, including Chicago, IL, Madison, WI, and each of the five boroughs of New York City. Broadly, we found that linking social capital is frequently tied to less COVID-19 spread (with Massachusetts as a contrarian case), while bonding and bridging social demonstrate mixed effects depending on the underlying social and racial histories of these communities.

In particular, we find that the effects of social capital are not uniform but divergent depending on whether those social ties link in-groups (bonding), bridge different groups (bridging), or engender trust in officials (linking), and the local context in which they are built.

### Contributions to the literature

**Figure 6:**
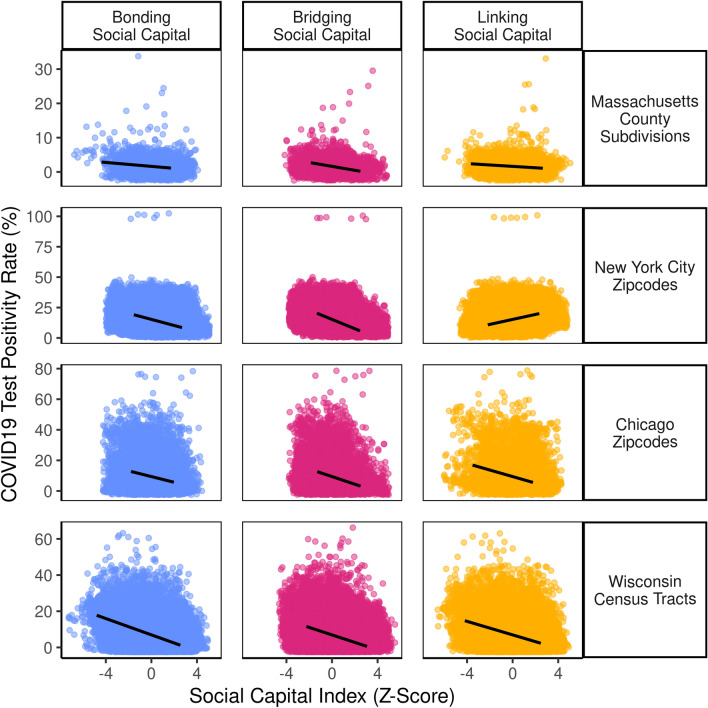
Social capital (mostly) reduces COVID-19 spread. Bivariate Scatterplots of social capital index scores compared to test positivity rates over, with lines of best fit depicting weak-to-strong negative associations for all except linking social capital in New York City.

Strong evidence was found supporting our hypothesis that social capital indices are strong, significant predictors of COVID-19 spread rates. Across counties, communities with stronger bonding social capital see lower rates of COVID-19 spread. Recent research on matched samples of US counties showed similar results, where bonding social capital seemed to help families and friends shelter and reinforced norms to wear masks and physical distance^22^. This largely confirms the trends found in past studies^10,12^, including those which use robust metrics like excess death rates^22^. It is worth noting that overall, when analyzed descriptively in Fig. 6’s scatterplots, each type of social capital was negatively correlated with COVID-19 spread.

The main exception in Fig. 6 is New York City, where linking social capital was related to worse spread. There are several reasons why this might be the case. First, Staten Island is home to 5% of New York City's population, but residents accounted for a quarter of COVID-19 fatalities in late 2020. The politically conservative borough has struggled with low resident participation in public health measures, including difficulty enforcing restrictions on indoor dining, residents refusing to wear masks^83^. And in Brooklyn, public health messaging has had difficulty penetrating some communities despite strong social ties. For example, tensions between Mayor Bill DeBlasio and the Orthodox Jewish community over COVID-19 have flared since March 2020, when the mayor sent police to break up a local rabbi's funeral and disperse crowds to prevent COVID-19 transmission. Such confrontations have been attributed to a decline in trust in public health measures and government response, and helped circulate rumors discouraging COVID-19 testing^84^.

However, after applying statistical controls, we found that each type of social capital produced varying effects depending on geography. When we zoomed into urban settings, bonding social ties took on mixed effects. In three out of six urban areas studied, communities with stronger bonding ties on average saw higher rates of COVID-19 spread, matching past findings in disaster studies, where bonding social ties *can* promote insular response^24^.

Further, bridging social capital showed varying effects, leading to lower spread in Massachusetts, Chicago, Brooklyn, the Bronx, and Wisconsin (although these last two effects had lower statistical significance, where p > 0.10). We theorize that bridging ties help ameliorate the deleterious effects of bonding social capital when present, but that since many bridging ties are formed in civil society groups and associations, they were occasionally the cause of early superspreader events. This study suggests that local planners should carefully inventory their community resources when responding to pandemics, in order to proactively channel bridging and linking social capital early into better public response.

### Limitations

Finally, this study came with several limitations. First, we relied on aggregate indicators sourced annually from the American Community Survey to make inferences about the state of social capital in communities. Future studies should ground-truth these measures by comparing them with survey responses in local communities. Second, this study also relied on a handful of indicators at the zipcode or county level, which were then averaged down onto census tracts, due to lack of availability. This primarily affected the bridging and linking indices, which fortunately still retain much variability within counties.

Third, as discussed in the “Methods” section, we relied on county averages to fill in some indicators of bridging social capital at the census tract level; future studies should identify comparable correlates or principal components to approximate such measures. Fourth, this study focused on data from 2010 to 2018, due to the limited availability of bridging and linking indicators, but future studies should extend this backwards to 2000.

Finally, this observational study showed associations between social capital and COVID-19 spread, but further studies are needed to verify whether these associations could be causal. Other factors may also influence COVID-19 spread depending on geographic context, including pollution^85,86^ and disaster damage^87^; natural and quasi-experiments may be helpful tools for further disentangling social capital’s effects from confounders^22^. Despite these limitations, these local level indices produce a considerably granular picture of the state of social capital in the US, far more so than previously available, and are a powerful tool for predicting the spread and abatement of COVID-19.

### Policy implications

In summary, this study analyzed the state of social capital in the United States between 2010 and 2018, generating estimates for bonding, bridging, and linking social capital in 72,877 census tracts over 9 years. Through geographic aggregation, we have produced estimates at the census tract, zipcode, and county subdivision level, which can serve as a resource for scholars and policymakers involved in disaster and pandemic response and recovery efforts. Further, this study identified through 3 regional studies and 6 urban case studies the considerable statistical power of these indices in predicting the spread of COVID-19.

We found considerable evidence that bonding social capital reduces COVID-19 spread at large, but especially in our urban case studies, this trend reversed. Instead, several cities displayed divergent trends, known as the Janus-faced nature of social capital^63^, where the insular, homophilous social networks fostered by bonding social capital were associated with greater COVID-19 spread (eg. Brooklyn, the Bronx, & Queens), while the bridging networks, trust, and reciprocity fostered by bridging social capital were associated with reduced COVID-19 spread in others cities (Madison, Manhattan, New York City at large, etc.),. Finally, this study found continued evidence that linking social capital is often negatively associated with COVID-19, matching past findings that trust in government and public health officials is key to pandemic prevention and response^22,64,71–73^. However, the positive association between linking ties and COVID-19 spread in Massachusetts hint that these trends are not set in stone and may fluctuate depending on local crisis conditions.

These findings highlight that while horizontal social ties usually aid in community resilience, local policymakers should pay special attention to encouraging trust and reciprocity among residents. This is especially vital considering that rising political polarization has been accompanied by pandemic denial from some state officials and rejection of social distancing and mask mandates among residents^49–51^.

Future studies should apply these indices to examine how social ties affected the movements and patterns of residents, economic activity, and physical and mental health during the pandemic—all important adjacent indicators of risk and resilience during the COVID-19 crisis. Further, scholars and policymakers should use these indices to identify communities as similar as possible to their own in terms of social capital in order to make projections about their communities’ recovery trajectories. Finally, scholars should apply these indices to diagnosing and explaining a wide variety of other community resilience, highlighting the close relationships between social capital and policy-relevant social outcomes, including health^16^, political polarization^49–51,88,89^, adaptation to climate change^90^, and resilience to future disasters^25,55,91^. We hope that these indices galvanize social capital scholars to examine the role of social capital in their communities’ recovery and to encourage greater attention to the role of residents and community planning in recovery and response to crisis.

## Methods

This study examines what kinds of communities experience greater outbreaks of COVID-19, measuring to what degree local-level measures of social capital and vulnerability predict those outbreaks. Below, we (1) outline our methodology for adapting these measures to the local level, and (2) describe our modeling procedures for case studies demonstrating the uses of these indices in predicting COVID-19 spread.

### Measuring social capital at the local level

To measure social capital, we took the average of three subindices (bonding, bridging, and linking) in each census tract, built out of 20 indicators total, listed in Table 1. These indicators were outlined and validated for the original county level social capital index^19^, but we describe them below as well.

**Table 1:** Indicators for census-tract level social capital indices.

Index	Concept	Indicator	Effect on index	Level	Years	Missing data (%)^a^	Literature
Bonding	Race similarity	Race fractionalization (0 = homogeneity, 1 = heterogeneity)	−	Tract	2010–18	0.9%	^92^
	Ethnicity similarity	Ethnicity fractionalization (0 = homogeneity, 1 = heterogeneity)	−	Tract	2010–18	0.9%	^92^
	Education equality	Negative absolute difference between % of residents with college education vs. did not graduate high school	−	Tract	2010–18	1.4%	^93,94^
	Race/income inequality	Gini coefficient (0 = equality, 1 = inequality)	−	Tract	2010–18	1.2%	^21^
	Employment equality	Absolute difference between % employed and unemployed labor force	** + **	Tract	2011–18	1.0%	^95^
	Gender income similarity^a^	Gender income fractionalization (0 = homogeneity, 1 = heterogeneity)	−	Tract	2010–18	3.4%	^68^
	Language competency	% Proficient English Speakers	** + **	Tract	2010–18	1%	^94^
	Communication capacity	% Households with telephone	+	Tract	2010–18	1.5%	^21^
	Non-elder population	% Below 65 years of age	+	Tract	2010–18	1%	^94^
Bridging	Religious Organizations	Religious organizations per 10,000 persons	+	Zipcode	2012–16	0.4%	^93^
	Civic Organizations^a^	Civic organizations per 10,000 persons Social Advocacy organizations per 10,000 persons	+	Zipcode	2012–16	0.7% (9.3%) 2.5% (22.0%)	^96^
	Social embeddedness—charitable ties^a^	Charitable organizations per 10,000 persons	+	Zipcode	2012–16	2.3% (20.7%)	^68^
	Social embeddedness—Fraternal ties^b^	Member of fraternal order (% of total)	+	County	2010	0%	^68^
	Social embeddedness—Union ties	Unions per 10,000 persons	+	Zipcode	2012–16	3%	^68^
Linking	Political Linkage	% of voting age citizens eligible for voting	+	Tract	2010–18	1%	^94^
	Local government linkage	% local government employees (per capita)	+	Tract	2010–18	1%	^97^
	State government linkage	% state government employees (per capita)	+	Tract	2010–18	1%	^97^
	Federal government linkage	% federal government employees (per capita)	+	Tract	2010–18	1%	^97^
	Political linkage-political activities^b^	% Attended political rally, speech, or organized protest	+	County	2010	0%	^95^

^a^Filled in missing census tract values with average value from census tracts in that county. Missing data tally reflects after imputing county median for specially marked zipcode bridging social capital measures.

^b^Used county level measure, because comparable measures were unavailable at local levels.

First, to represent bonding social capital, we use 9 indicators describing how similar residents in a community are in terms of race, age, class, gender, language, and communication capacity, because homophilous communities tend to have strong bonding social ties^98,99^. To represent *race similarity*, we used a fractionalization approach to measure how fractionalized a community is into different racial categories, where 0 represents homogeneity and 1 represents heterogeneity^92^. We repeated this approach for *ethnicity similarity*, using the share of residents which identified as Latino or Hispanic, or not^92^, as well as *similarity between genders by income*^95^. To represent *educational equality*, we calculated the negative absolute difference between the share of residents with a college education compared to those which did not graduate high school^93^. Each of the aforementioned negative measures were then reverse-coded, so that a low value denotes low heterogeneity while high values denote homogeneity. Finally, we measured four more positive measures of homogeneity. To represent *employment equality*, we calculated the absolute difference between the share of the employed and unemployed labor force, because this indicates that most of the labor force is similarly employed^96^. To represent *language competency*, we used the percentage of residents who speak English proficiently; to represent *communication capacity*, we used the share of households with a telephone; while to represent age, we used that share of residents below 65 years of age^21,94^.

Second, to represent bridging social capital, we use 6 indicators of membership in associations that can bridge different parts of society. We measured the number of *religious organizations*^93^, *civic organizations*, *social advocacy organizations*^96^, *charitable organizations,* and *unions*^68^, normalized per 10,000 residents. Each of these was obtained from the Zipcode Business Patterns census, and then averaged to the census tract level. Finally, we supplement this with one county tally not available at the zipcode level, the percentage of residents participating in a *fraternal order*.

Third, to represent linking social capital, we use 5 indicators of connection and representation. To represent *political linkages*, we gathered the share of voting age eligible citizens^94^, while to represent *government linkages*, we measured the share of *local*, *state*, and *federal* government employees per capita^97^. We supplemented these census tract level measures with one county level measure of *political activities*, namely the share of residents who attended a political rally, speech, or organized protest that year^95^.

This culminated in four indices, including bonding, bridging, and linking social capital, as well as overall social capital, which represents the average of its three subindices. To combine indicators, we use the mean, because this best approximates the midpoint of a small number of indicators for a given census tract. This is better compared to the median, which could potentially completely ignore the influence of the lowest indicator in favor of more commonly occurring values (for an example, see Fig. A1). Each index was measured at the census tract level, and their distributions are visualized across US Census Divisions in Fig. 1 to demonstrate their degree of geographic variation.

This data comes with several caveats. The indicators for these indices, presented in Table 1, are based on various tallies pre-aggregated by the census to the lowest level at which they would provide these data (eg. tract, zipcode, or county subdivision). It was not possible to test the geographic distributions of these tallied units (eg. people or organizations) within a census tract, zipcode, or subdivision; that would require individual records for each organization, but the census does not provide these, so as to preserve the privacy of respondents. These indices cannot explore spatial variation within a community; as such data becomes available, we encourage future studies to test such questions. Fortunately, they are well suited to expose spatial variation in social capital among communities, as shown below in Fig. 1, and later, in Fig. 2.

We present the distributions of our indicators using density plots in Appendix Fig. A2; indicators largely retained a bell curve shape, except for a handful, such as language competency and communication capacity. However, we view this as important evidence of variation in aspects of bonding social capital; it is important to up- or downweight- communities’ bonding indices based on these observed trends, since such factors are closely involved in building in-group ties. The exception was for rates, including all bridging social capital indicators (organizations normalized by population) and 3 linking indicators (government employees normalized by population). Because rates are always right-skewed, we log-transformed these (before rescaling from 0 to 1) to be more comparable to other variables. Finally, to deal with outliers, while retaining the general shape of these distributions, we capped all indicators at their 2.5th and 97.5th percentiles.

### Missing data

While 15 out of 20 indicators were missing 1% of data points or less, 3 indicators were missing considerable amounts of data. These variables included social advocacy groups (missing 22%), charitable organizations (20.7%), and civic organizations (9.3%). While complete data is obviously preferable, these associational measures are vital to the measurement of bridging social capital. Despite these missing data points, most of these census tracts were surrounded by other census tracts where this data was widely available. As a result, we use a two-stage imputation process, first imputing missing data for these five variables alone with each census tract’s county median value. This reduces missing data considerably, and ensures that our indices are geographically consistent. After county median imputation, social advocacy groups lack only 2.5% of data points, charitable organizations lack just 2.3%, and civic organizations lack just 0.7%. These are much better levels of missing data.

To fill in remaining data points, we leverage the statistical power of our dataset of 72,877 census tract observations from 2010 to 2018, using multiple imputation using the Amelia II software (version 1.76) in R (version 4.0.3)^100^. Using time-series imputation and melding results across 5 imputed datasets improves imputation for missing data over time, because it fills in data points from, for example, 2013, by taking into consideration sensible values given that census tract’s values in 2012 and 2014. While complete data is preferable, this two-handed strategy helps keep missing data points geographically similar to those in their county and temporally similar to observations of the same census tract over time. Our indices reflect local level variation considerably better than would a local level index that *excludes* these indicators.

### Aggregation

Finally, we aggregated these census tract measures to the zipcode and county subdivision levels as well. Since these geographies do not overlap perfectly, for each zipcode or county subdivision, we identified all census tracts that overlap with these jurisdictions and took the median index score for each index, which best captures the most likely index score for that tract given its surroundings. We repeated this process with the CDC's 2018 Social Vulnerability Index^20,101^ and its four subcomponents, to enable comparisons between social capital and social vulnerability indices at the census tract, zipcode, and county subdivision levels.

### Variables

Finally, to demonstrate the value of these granular social capital indices, we modeled the effects of social capital on COVID-19 spread in several case studies, to be discussed further below. This study uses the following variables to represent key factors in COVID-19 spread.

As our outcome variable, we used the test positivity rate—*the percentage of tests that return positive*. This is a useful outcome to measure, because it highlights how widespread infection is in a community, while controlling for the amount of testing in a locale^74^. In contrast, straight case rates and death rates may miss unidentified cases. (Other ideal measures, such as excess deaths rates, are not currently available below the county level). Test positivity rates are a useful proxy for the spread of COVID-19, because these rates closely correlate with COVID-19 case rates, rates of residents with COVID-19 antibodies^77^, hospital admissions rates^75^, and death rates^76^. Rates of positive tests have been used to measure spread in studies in US counties^102^, Louisiana county subdivisions^87^, and New York City zipcodes^78,79^, among others.

Since this outcome is right skewed, we log transformed it and applied linear models, as discussed below. Because our outcome is a percentage, these models already adjust for the size of the population.

For each case study, we tested the effects of bonding, bridging, and linking social capital on test positivity rates, controlling for the CDC's for 4 social vulnerability sub-indices. These include (1) *socioeconomic status*, (2) *minority status and language*, (3) *household composition and disability*, and (4) *housing type and transport*^20,94^.

Additionally, we controlled for population mobility, measured over time at the county level, using the average daily change in workplace mobility, estimated with Google android user movement^103^. These measures have been frequently used to study COVID-19 and mobility (eg.^11,51,62^). We averaged movement between two and eleven days prior to each observation, since 95% of individuals manifest symptoms between 2 and 11 days after contact with COVID-19^31–33^.

Finally, we also controlled for several other constant county level traits. These include *health care capacity*, which we measured by averaging two rescaled indicators, including the number of primary care physicians per 100,000 residents in 2017^36^ and the number of preventable hospital stays per 100,000 residents in 2017, which we reverse scaled^104^. We also measured *overall quality of health* by averaging seven indicators. These include the precentage of residents identified as current smokers in 2017, drinking excessively in 2017, who reported being physically inactive in 2016, who had diabetes in 2016, who were obese in 2016, who reported experiencing poor physical health over 14 days in a month in 2017, and the age-adjusted premature mortality rate (deaths under age 75) between 2016 and 2018. These measures were gathered from the County Health Rankings^105^. We also control for governance capacity, measured by the number of municipal employees per capita from the American Community Survey in 2018, and for partisanship, measured by the share of residents who voted for the Democratic presidential candidate in 2016 using data from the *MIT Elections Lab*^106^.

### Case studies

We applied this array of variables to two types of case studies, including (1) cases *across counties* and (2) cases *within counties.* These cases were located in the Northeast, Mid-Atlantic, and Midwest, to highlight indices in different regions and geographic levels, where COVID-19 outcome data had been reported at these granular levels. (While it is rare for entire states to report these outcomes, many cities do).

First, we examine variation *across counties* at our three levels of interest, drawing from (1) weekly test positivity rates over a 7-day period in 1393 Wisconsin census tracts from April 6, 2020 to November 16, 2020, (2) daily test positivity rates over a 7 day period in 178 New York City zipcode tabulation areas within the five boroughs from May 18 to October 28, 2020, and (3) weekly test positive rates over a 14 day period in 350 Massachusetts county subdivisions from September 30 to November 19, 2020.

Massachusetts, Wisconsin, and New York City used the rate of molecular tests (PRC tests)^107–109^, while Chicago’s test positivity rates combine both molecular and antigen test results^110^. Despite including antigen testing, Chicago retained the same negative associations for bridging and linking social capital found in Wisconsin, Madison, Manhattan, Queens, and New York City, although Chicago’s trends demonstrated lower statistical significance (p-values = 0.30–0.70) (see “Results”). Further, it is worth noting that the study period (2020) largely predates mass-availability of antigen tests in local convenience stores, so during this period, we expect that molecular tests reflect well the state of testing across different cities.

For these cases, we model the effect of social capital indices, controlling for social vulnerability indices, at the census tract, zipcode, or county subdivision, and we then control for county-level traits including mobility, health care capacity, overall health quality, governance capacity, and partisanship.

Our primary goal in these models is to evaluate and compare the effects of bonding, bridging, and linking social capital indices across different geographic contexts. In contrast, some differences between control variables’ effects, eg. mobility or governance capacity, are to be expected, due to different levels of governance capacity and COVID precautions (although shared effects among control variables are always a good sign). To test the consistency of social capital effects, in Table B2, we break down our original Table B1 models *across* counties, showing to what degree the effects of social capital indices remain *consistent* when using just our independent variables with no controls (Models 1A, 2A and 3A), basic controls (Models 1B, 2B, and 3B), and full controls (Models 1C, 2C, and 3C, the same as Models 1–3 in Table B1). In the results, we report associations which persist across each level of controls, as a validity check to ensure our results are not simply due to model specification.

Then, for each model, we compared fixed effects by date, random effects by jurisdiction, and random effects by jurisdiction nested within counties. The advantage here is that by nesting within multiple counties, we can control for county wide traits. We used Hausman tests to choose between fixed, random, and nested random effects. Given a statistically significant Hausman test (in this case, p < 0.001), we reported fixed effects, and otherwise reported nested random effects, which fit better than random effects alone. Models tables are presented in the Supplementary Information.

Second, we examine variation *within counties* at the zipcode level and census tract level (there are usually too few county subdivisions to do this otherwise). We draw from 77 census tracts in Madison, WI from April 6, 2020 to November 16, 2020, from 60 zipcodes in Chicago, IL from March 7 to November 14, 2020, and from 44 zipcodes in Manhattan, 25 in the Bronx, 37 in Brooklyn, and 60 in Queens between May 18 and October 28, 2020. (Staten Island could not be modeled individually due to considerable collinearity between multiple social capital and vulnerability indicators). Each model includes social capital and social vulnerability indicators, with fixed effects by date or random effects by jurisdiction. Since county variables are time-invariant or correlate with temporal fixed effects, they cannot be included in these models, which are intended to use as few variables as possible, to demonstrate the considerable predictive power of these main indices together. Significant Hausman tests (in this case, p < 0.001) led us to use fixed effects for models of Madison, Brooklyn, and Manhattan, while results of limited statistical significance (p ~ 0.90) led us to use random effects for Chicago, the Bronx, and Queens. These are visualized in Fig. 4, using marginal effects^111^ calculated in the *ggeffects* package (version 1.1.1) in R (version 4.0.3)^114^, as described in the “Results”.

Finally, we repeated all nine models (*within counties* and *across counties*) as simple OLS models for each timestep to demonstrate the high predictive power of social capital and controls in each timestep. We use the R^2^ statistic, the percentage of variance explained in the outcome, to represent this, and plot this over time for each sample in Fig. 3.

### Goodness of fit and validity

Figure 3 required generating hundreds of models in a loop, but each of these small OLS regression models fulfilled necessary assumptions^112^: First, no model demonstrated problematic collinearity in these models; all variance inflation factor scores were below 10, the threshold for problematic collinearity. Second, there was no problematic heteroskedasticity; by modeling the data separately for each time period, we removed any temporal relationships; further, heteroskedasticity affects standard errors, but not model-level statistics like the R^2^ statistic, remedying any such concerns.

Further, in our reported models in Tables B1–B3, several models had highly collinear variables, usually between social capital and social vulnerability variables. We performed a series of transformations on these variables, which reduced collinearity for each variable, measured by the variance inflation factor, to below 10, the indicator for problematic levels, and near 2.5, the gold standard. In a handful of cases, problematic covariates like governance capacity were dropped when transformations would not suffice. These steps are described beneath each Supplementary Information table. Results of these models are presented above in the main text, while model tables are listed in Tables B1–B3.

### Ethics declarations

This study involved no human subjects and relied on only aggregate, publicly available data and therefore did not require ethics review.

## Supplementary Information

Supplementary Information.

## Data Availability

All code necessary for replicating this study will be made available for replication on the Harvard Dataverse (https://doi.org/10.7910/DVN/OSVCRC).
