# Supplementary material for: Social capital's impact on COVID-19 outcomes at local levels
Source: Sci Rep. 2022 Apr 21;12:6566. doi: 10.1038/s41598-022-10275-z (PMC9022050; doi:10.1038/s41598-022-10275-z)
Supplement: Supplementary file 1 — Supplementary Information. [file 41598_2022_10275_MOESM1_ESM.docx]

**Supplementary Information Appendix**

for manuscript:

**Social Capital's Impact on COVID-19 Outcomes at Local Levels**

### **Table of Contents**

**Appendix A: Indices (Continued)**

- **Figure A1**: Calculation of Index Score for 5 indicators for Hypothetical Census Tract
- **Figure A2:** Distributions of Indicators

### **Appendix B: Modeling (continued)**

#### **Table B1: Modeling Social Capital with Controls in Massachusetts, New York, & Wisconsin**

#### **Table B2: Effect of Social Capital given different levels of controls in Massachusetts, New York, & Wisconsin**

#### **Table B3: Modeling Social Capital with Controls within Counties**

### *Data availability:* All code necessary for replicating this study will be made available for replication on the Harvard Dataverse (<https://doi.org/10.7910/DVN/OSVCRC>).

**Appendix A: Indices (Continued)**

**Figure A1: Calculation of Index Score for 5 indicators for Hypothetical Census Tract**



**Figure A2: Distributions of Indicators**

**
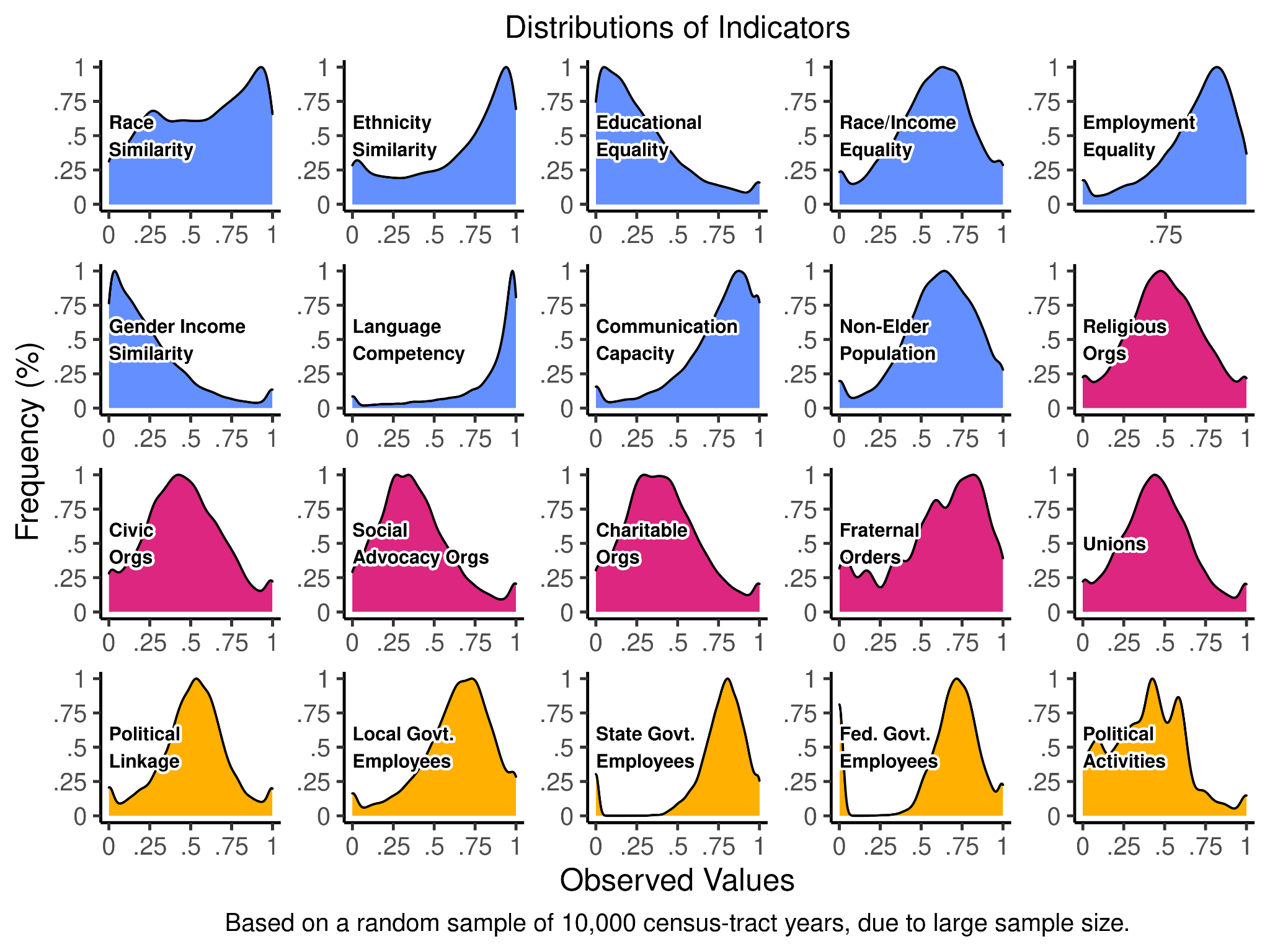
**

###

#### **Table B1: Modeling Social Capital with Controls in Massachusetts, New York, & Wisconsin**


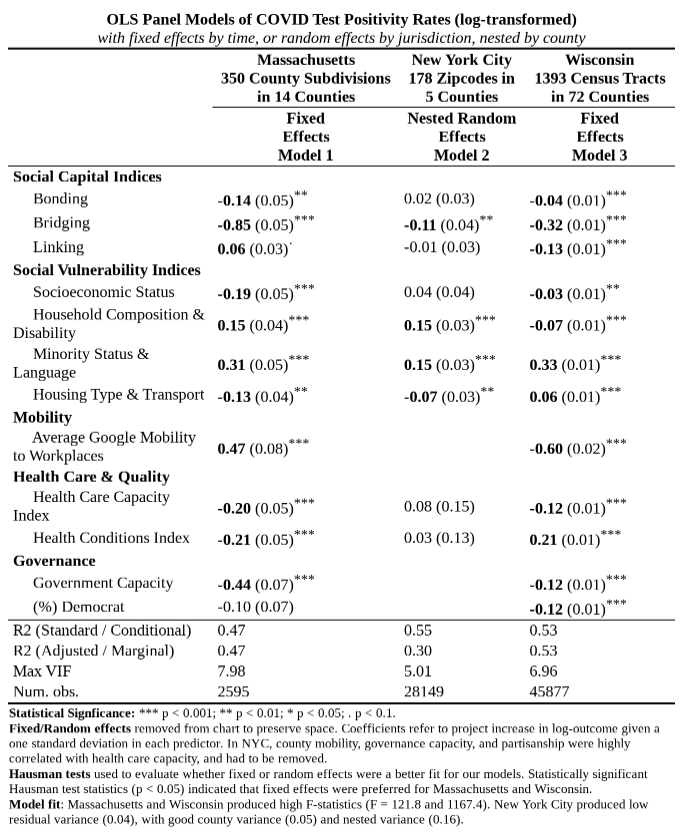


**Note on Table B1:** This analysis required several transformations to remove collinearity. For Massachusetts models, we log-transformed our index for Socioeconomic Status. For New York City models, socioeconomic status and minority status & language were log-transformed. Further, health care capacity was strongly correlated with mobility, government capacity, and partisanship, and the latter three had to be removed to reduce collinearity. Finally, for Wisconsin models, no transformations were necessary.

#### **Table B2: Effect of Social Capital given different levels of controls in Massachusetts, New York, & Wisconsin**


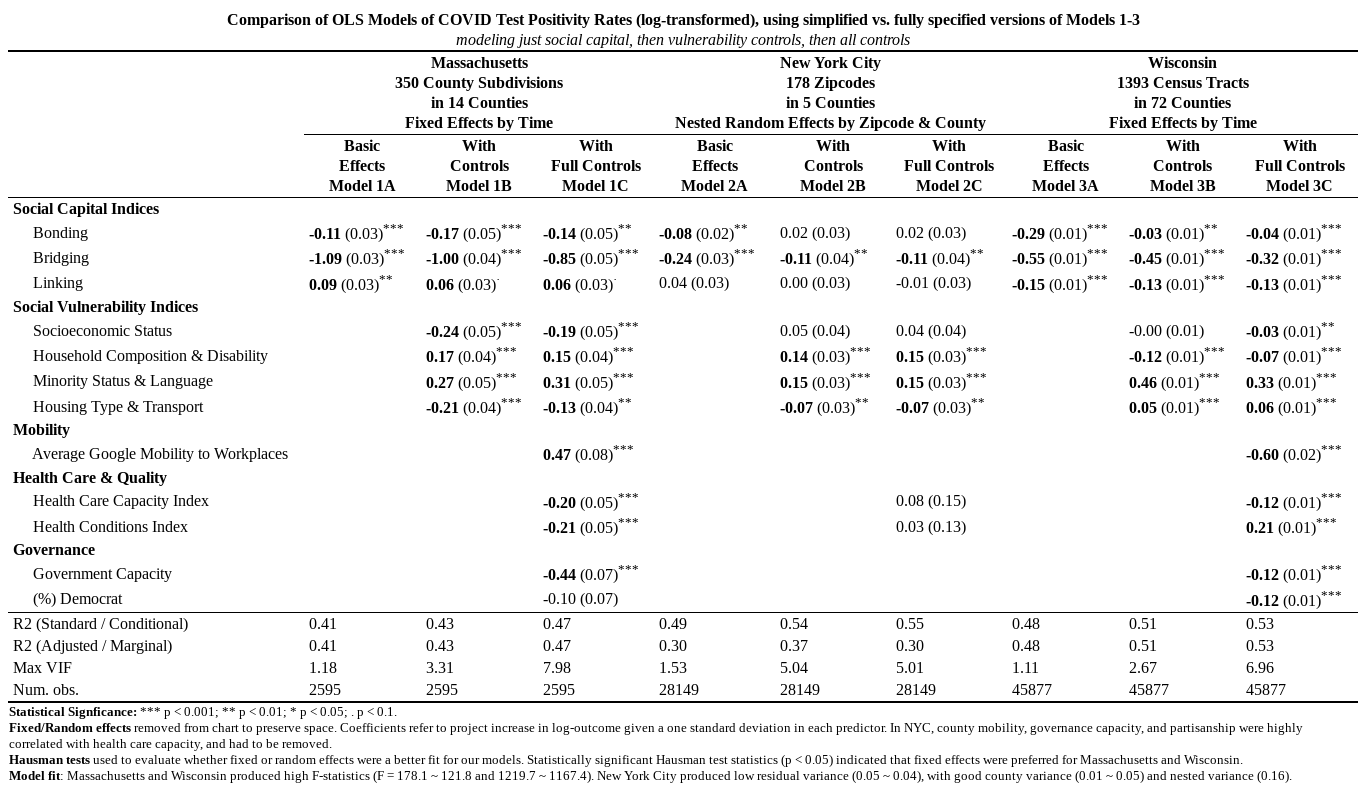


**Note on Table B2:** Further, we break these best-fitting models from **Table B1** down to show just the effect of social capital, then with vulnerability controls, and finally with all controls. These help clarify that the social capital effects we find statistically significant in our final analysis are not statistical noise but rather consistent effects across modeling strategies.

#### **Table B3: Modeling Social Capital with Controls within Counties**


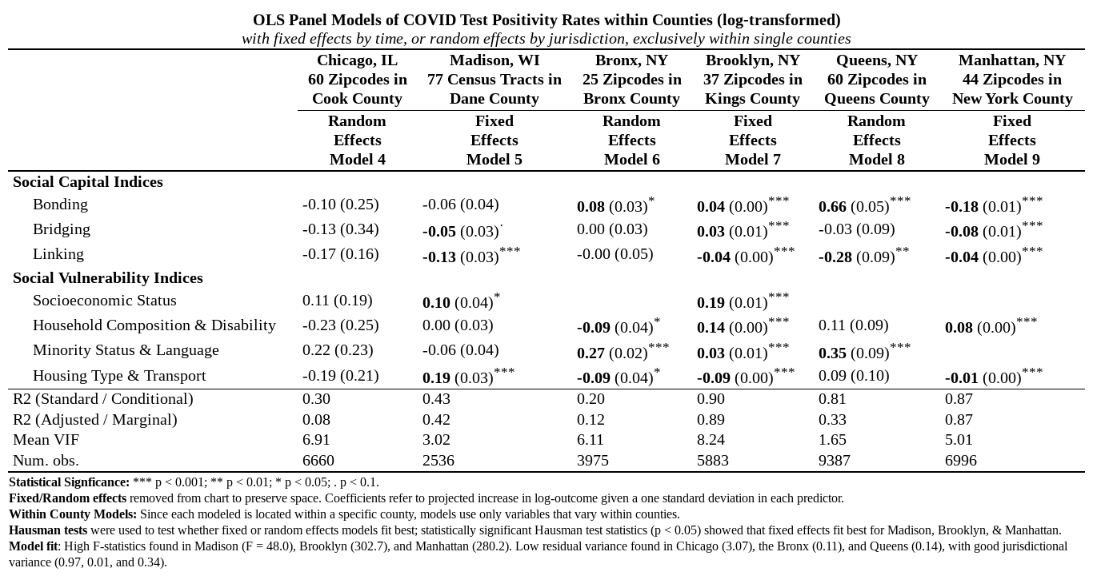


**Note on Table B3:** This analysis also required several transformations to remove collinearity, particularly Chicago models. Here, we squared socioeconomic status, took the reciprocal transformation of household composition and disability, took the reciprocal transformation of the square of minority status, and took the log of bonding social capital. For the Bronx, we dropped socioeconomic status and broke minority status into three quantiles and squared it to break the association. (It was important to still control for vulnerability by race and ethnicity in the Bronx.) In Brooklyn, we took the log of socioeconomic status. In Queens, we took the log of socioeconomic status and minority status, while in Manhattan, we broke bonding social capital into six quantiles and squared it.While such heavy transformations are generally to be avoided, they were necessary to reduce strong collinearity between bonding social capital, minority status, and socioeconomic status, which are deeply intertwined in American cities.
